# Application of RFLP-PCR-Based Identification for Sand Fly Surveillance in an Area Endemic for Kala-Azar in Mymensingh, Bangladesh

**DOI:** 10.1155/2012/467821

**Published:** 2012-05-31

**Authors:** Mohammad Shafiul Alam, Hirotomo Kato, Mizuho Fukushige, Yukiko Wagatsuma, Makoto Itoh

**Affiliations:** ^1^Parasitology Laboratory, International Center for Diarrhoeal Disease Research, Bangladesh (ICDDR,B), 68 Shaheed Tajuddin Ahmed Sarani, Mohakhali, Dhaka 1212, Bangladesh; ^2^Laboratory of Parasitology, Department of Disease Control, Graduate School of Veterinary Medicine, Hokkaido University, Sapporo 060-0818, Japan; ^3^Department of Epidemiology, Graduate School of Comprehensive Human Sciences, University of Tsukuba, Ibaraki, Tsukuba 305-8577, Japan; ^4^Department of Parasitology, Aichi Medical University School of Medicine, Nagakute 480-1195, Japan

## Abstract

Mymensingh is the most endemic district for kala-azar in Bangladesh. *Phlebotomus argentipes* remains the only known vector although a number of sand fly species are prevalent in this area. Genotyping of sand flies distributed in a VL endemic area was developed by a PCR and restriction-fragment-length polymorphism (RFLP) of 18S rRNA gene of sand fly species. Using the RFLP-PCR analysis with *Afa*I and *Hin*fI restriction enzymes, *P. argentipes, P. papatasi*, and *Sergentomyia* species could be identified. Among 1,055 female sand flies successfully analyzed for the species identification individually, 64.4% flies was classified as *Sergentomyia* species, whereas 35.6% was identified as *P. argentipes* and no *P. papatasi* was found. Although infection of *Leishmania* within the sand flies was individually examined targeting leishmanial minicircle DNA, none of the 1,055 sand flies examined were positive for *Leishmania* infection. The RFLP-PCR could be useful tools for taxonomic identification and *Leishmania* infection monitoring in endemic areas of Bangladesh.

## 1. Introduction

Phlebotomine sand flies are blood-sucking insects belonging to the family Psychodidae in the order Diptera [1]. Hitherto, approximately 800 sand fly species have been recorded; however, the majority of the species play no part in the transmission of leishmaniasis in nature, and less than 10% of sand flies has been incriminated as vector species of leishmaniasis [1–3]. In addition, each vector species can only support the development of specific *Leishmania* species and consequently can only transmit certain species of the genus [2]. Thus, surveillance of prevalent sand fly species and *Leishmania* infection within sand flies in each endemic area is important for prediction of the risk of transmission and expansion of the disease.

Kala-azar (visceral leishmaniasis: VL) caused by *Leishmania donovani* complex is the most severe form of leishmaniasis and the second-largest parasitic killer in the world with an estimated 500,000 new cases and more than 50,000 deaths each year. More than 90% of the world's cases of VL is in India, Bangladesh, Nepal, Sudan, and Brazil, affecting the poorest of poor people [4]. *Phlebotomus argentipes* has been implicated as the only vector for VL in the north-east Indian subcontinent (part of India, Bangladesh, Nepal) [5, 6].

In Bangladesh, Mymensingh district is the most highly endemic areas of VL and accounted for more than 50% of the total cases reported from 2000 to 2004 [7]. However, except for one particular instance [8], information on distributing sand fly species and vector species responsible for the disease transmission is scarce despite its potential importance for the disease and vector control in this area.

Taxonomic identification of sand fly species except for some male species (terminal genitalia) mostly based on internal characteristics of females (cibarium, pharynx, and spermatheca) [6, 9]. Thus, identification of sand fly species requires specific knowledge and skills. In recent time there is a lack of skilled entomologist in Bangladesh and at the same time new generations are not interested to take this subject as a profession due to some coherent reasons [10]. Under the circumstances a molecular approach to identify the sand fly species will help the country to plan their control program successfully.

In the present study, (1) genotyping of sand flies distributing in the VL endemic area was developed by a RFLP-PCR of 18S rRNA gene, (2) sand flies caught in the area were identified with the RFLP-PCR and infection status of sand fly species by the *Leishmania* was also examined.

## 2. Materials and Methods

### 2.1. Collection of Sand Flies

Sand fly collection was made from May to July 2008 in Trishal of Mymensingh district, Bangladesh. The sand flies were captured in houses from using CDC miniature light trap (Model 512, John W. Hock Company, FL, USA). The traps were set at 4:00–7:00 p.m. and collected at 6:00–9:00 a.m. in the next morning. The sand flies were kept in 70% ethanol and stored at room temperature until further use. A total of 6,123 sand flies were collected from 169 houses in single night collection.

### 2.2. Morphological Identification of Sand Flies

Polyvinyl alcohol (PVA) mounting medium (BioQuip Products, Inc, Los Angeles, CA, USA) was used for slide preparations of morphological classification, and 1,712 flies were identified under a microscope with the keys developed by Lewis [11]. Based on internal and external morphological characteristics, *Phlebotomus argentipes * and two *Sergentomyia *species were identified in this pool [12]. Another ethanol-fixed 2,708 flies were examined under a stereomicroscope, and 1,342 (49.6%) female flies were subjected to a genotyping method [12].

### 2.3. Genotyping by RFLP-PCR

DNA extracts from sand flies morphologically identified as *P. argentipes, P. papatasi,* and two *Sergentomyia *species were analyzed by RFLP-PCR. PCR was carried out with Lu.18S 1S and Lu.18S AR primers and Lu.18S 1S and Lu.18S 1R primers with which amplified approximately 2,000 bp and 450 bpb fragments of sand fly 18S rRNA gene, respectively [13–16]. The amplicons were digested by *Afa*I or *Hin*fI restriction enzyme.

### 2.4. DNA Extraction from Individual Sand Flies

Extraction of DNA from individual sand flies was performed using 96-well U-bottom plates [13–16]. Briefly, ethanol-fixed sand flies were placed individually in each well of 96-well plates, dried at least for 1 hour at room temperature to evaporate ethanol, and lysed in DNA extraction buffer without homogenization. The samples were incubated at 37°C for 12 hours and directly used as the templates for Ampdirect PCR without purification process such as phenol/chloroform extraction. DNA samples from 1,342 field caught female sand flies were subjected to RFLP-PCR analysis for the species identification. Infection of *Leishmania* within the sand flies was individually examined targeting leishmanial minicircle DNA [13–16]. The method is sensitive enough to detect only one *Leishmania* parasite within individual sand flies [13, 14].

## 3. Results

**Figure 1:**
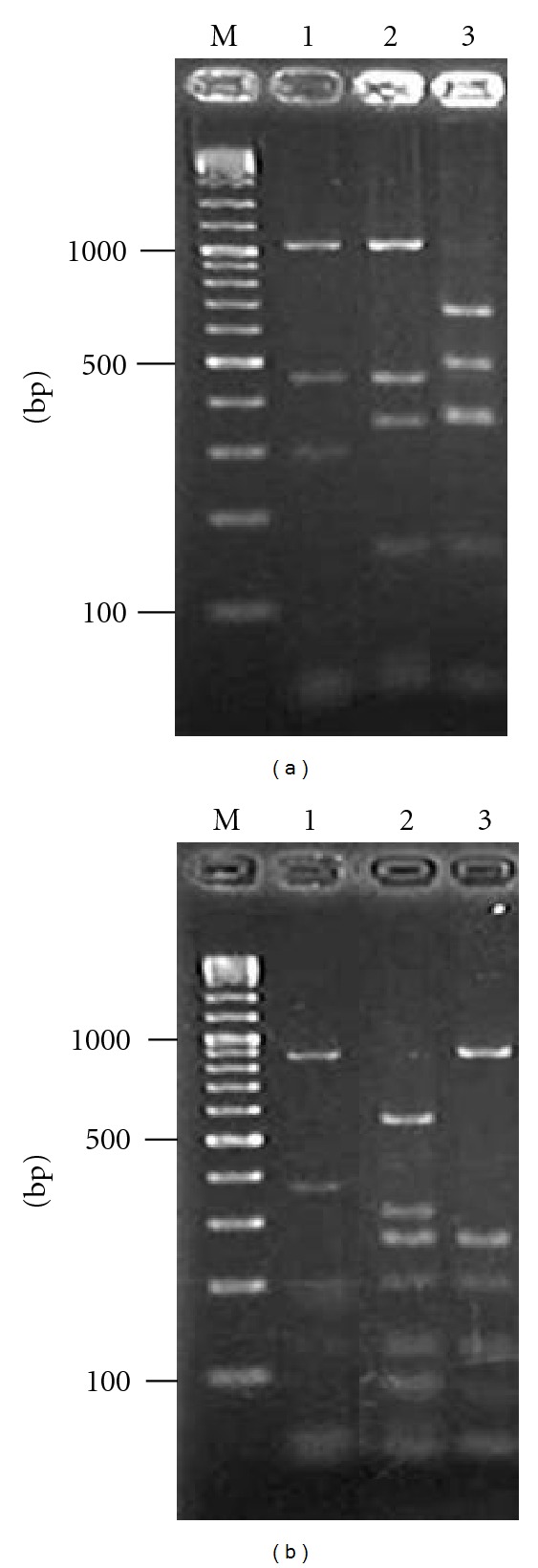
RFLP-PCR analyses of 18S rRNA genes of *Phlebotomus (P.) argentipes* and two *Sergentomyia* species. PCR amplification with Lu.18S 1S and Lu.18S AR primers was performed to amplify approximately 2,000 bp fragments of sand fly 18S rRNA gene. The PCR products were digested with *Afa*I (a) or *Hin*fI (b), and the digested samples were separated by electrophoresis in a 2% agarose gel to produce DNA fragments. Lane M; 100-basepair ladder, lane 1; *P. argentipes*, lane 2; *Sergentomyia *species A, and lane 3; *Sergentomyia *species B.

**Figure 2:**
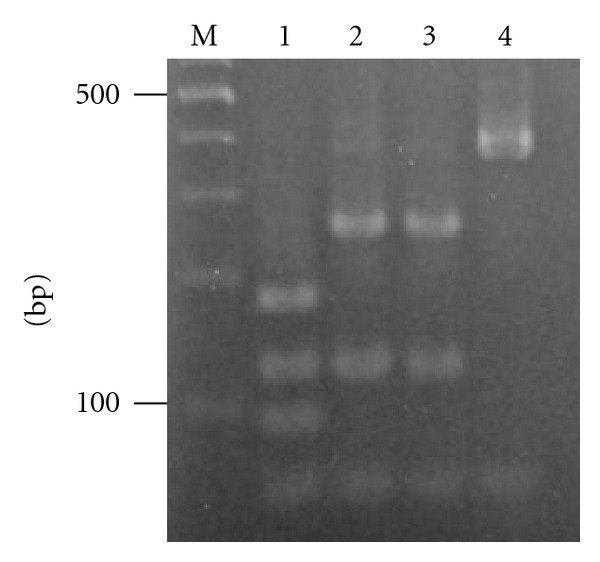
RFLP-PCR analyses of 18S rRNA genes of* P. argentipes*, two *Sergentomyia* species, and *P. papatasi*. PCR amplification with Lu.18S 1S and Lu.18S 1R primers was performed to amplify approximately 450 bp fragments of sand fly 18S rRNA gene. The PCR products were digested with *Hin*fI, and the digested samples were separated by electrophoresis in a 3% agarose gel to produce DNA fragments. Lane M; 100-basepair ladder, lane 1; *P. argentipes*, lane 2; *Sergentomyia *species A, and lane 3; *Sergentomyia *species B. Lane 4;* P. papatasi. *

The RFLP patterns of morphologically identified *P. argentipes * and two *Sergentomyia *species, A and B, were analyzed on the 18S rRNA fragments of the 2,000 bp. The three species were clearly differentiated by single digestion with *Afa*I or *Hin*fI restriction enzyme (Figures 1(a) and 1(b)). On the other hand, RFLP analysis of the 450 bp amplicons with* Hin*fI differentiated *P. argentipes, P. papatasi* from *Sergentomyia* species, although two *Sergentomyia* species were indistinguishable (Figure 2). As preliminary study of the analysis showed that the 2,000 bp target was difficult to be amplified in part of the sand fly samples, the RFLP-PCR analysis of the 450 bp amplicon was employed for further genotyping of the samples from the field. The less efficiency for the amplification of longer fragments is probably associated with the postmortem changes that may occur in dead specimens in the light trap.

DNA samples from 1,342 field caught female sand flies were subjected to RFLP-PCR analysis for the species identification. Although DNA fragment was not amplified in 287 samples, among the 1,055 samples amplified, 679 flies (64.4%) were classified as *Sergentomyia* species whereas 376 (35.6%) were identified as *P. argentipes*. No sample was identified as *P. papatasi*. Although infection of *Leishmania* within sand flies was individually examined targeting leishmanial minicircle DNA, none of the 1,055 sand flies examined were positive for *Leishmania* infection.

## 4. Discussion

In the present study, genotyping of sand flies was successfully developed for differentiating the sand fly species in VL endemic area in Mymensingh, Bangladesh. Sand flies morphologically identified as *P. argentipes*, *P. papatasi, *and *Sergentomyia *species could be differentiated by the RFLP-PCR using amplicons of 450 bp. Further differentiation of *Sergentomyia *species A and B was made by the method using amplicons of 2,000 bp. Using the present method, more than a thousand of sand flies could be examined in a short time for their species and presence of *Leishmania* parasite.

The major sand fly species detected in this study, *P. argentipes* and *Sergentomyia* spp., were same with the previous morphological observation made with sand flies collected from a whole areas in the same district [8, 12].

*P. argentipes* is the proven vector of *Leishmania (Leishmania) donovani* in India [17]. Although *Sergentomyia *species mainly feed on cold-blooded vertebrates, such as, reptiles [11], transmission of VL by *Sergentomyia *species may be taken into account since a previous study suggested possible infection of *Sergentomyia *species with *Leishmania* species [18]. Another *Phlebotomus* species, *P. papatasi*, which is the proven vector of *L. (L.) major* causing cutaneous leishmaniasis (CL), was morphologically identified in other parts of Bangladesh [19]. The species could be differentiated by the present RFLP-PCR method and was not found in the present sand fly pool. The absence of *P. papatasi* in Mymensingh district was also reported earlier [8].

Natural infection of *Leishmania* species among sand flies collected was not detected in this study. It was probably because the infection rate is usually very low (0.01–1%) among sand fly populations even in endemic areas [3]. Further surveillance of larger populations with the present mass screening method will provide more information on the endemic area.

In conclusion, the sand fly species reported in a VL endemic area in Mymensingh Bangladesh could be identified by RFLP-PCR and the present method could be a useful tool for identification of species and examination of *Leishmania* parasite infection of large number of field sand fly samples.
